# Development of an intravaginal ring for the topical delivery of Aurora kinase A inhibitor, MLN8237

**DOI:** 10.1371/journal.pone.0225774

**Published:** 2019-11-27

**Authors:** Yaman Tayyar, Ryan Shiels, Andrew C. Bulmer, Alfred K. Lam, Daniel Clarke, Adi Idris, Nigel A. McMillan

**Affiliations:** 1 School of Medical Science, Griffith University, Southport, Australia; 2 Menzies Health Institute Queensland, Griffith University, Southport, Australia; Institut de Genetique et Developpement de Rennes, FRANCE

## Abstract

Human papilloma virus (HPV) is the main culprit in cervical cancers. Although the HPV vaccine is now available, the slow and gradual process for HPV cancers to form means little will change, even for vaccinated individuals. This warrants the development of new therapeutic strategies in both the newly diagnosed and recurrent patients. We have previously shown that Alisertib (MLN8237), an Aurora A kinase inhibitor, potently and selectively kills HPV-positive cervical cancer cells. However, Alisertib is known for its unfavorable side effects when administered systemically. A targeted delivery approach is therefore warranted. The topical delivery of drugs to the cervix for the treatment of cervical cancer is an underexplored area of research that has the potential to significantly improve therapeutic outcome. Here, we design a novel topical drug delivery system for localized delivery in the vaginal tract using intravaginal silicone rings loaded with Alisertib. We assessed the suitability of the drug for the application and delivery method and develop a high-performance liquid chromatography method, then show that the vaginal rings were effective at releasing Alisertib over an extended period of time. Furthermore, we showed that Alisertib-loaded vaginal rings did not induce overt inflammation in the mouse vaginal tract. Our work has major translational implications for the future development of vaginal ring devices for the topical treatment of cervical cancer.

## Introduction

Persistent infection with human papillomavirus (HPV) is the key risk factor for cervical cancer and is found in more than 99% of cervical cancer cases [1, 2]. Current treatments for cervical cancer involve a combination of surgery, radiotherapy, and/or chemotherapy. However, little improvement in patient outcomes has occurred with these treatments over the last 35 years [3]. We recently showed that Aurora A kinase inhibition using Alisertib (MLN8237) was selectively lethal for HPV-positive (+) cervical cancer cells, both *in vitro* and *in vivo* [4, 5]. Given that more than 75% of all cervical cancer cases are diagnosed within stages I–II [6, 7], where the cancer is still confined to epithelia of the cervix itself, it was rational to explore a potential localized delivery system for administering Alisertib in the cervical area. Intravaginal rings (IVRs) represent a topical delivery system for female genital disorders, that sit in the cervical area when administered [8]. IVRs are flexible, torus in shape and elastomeric drug carriers that maintain long-term delivery of a range of drugs currently in use, including microbicides, contraceptives and anti-HIV agents with demonstrated superior efficacy and convenient release profiles [9–16]. IVR polymers are commonly hydrophobic in nature and therefore provide a compatible carrier for hydrophobic molecules [17], such as Alisertib. Although local delivery of therapeutic drugs for cervical cancer may provide higher efficacy and better safety profile, little is known about the potential of IVRs in delivering Alisertib, let alone in the cervix. In this study, we explored the possibility of developing a topical delivery platform of Alisertib for the application in the vaginal tract. Matrix type silicone based IVRs were chosen as a starting point due to their compatibility with Alisertib, their safety profile, and expected favourable release profile. To our knowledge, this is the first demonstration of localizing the administration of Alisertib into the vaginal tract.

## Materials and methods

### Cells

The HPV+ cervical cancer cell line, CaSki, was obtained from the American Type Culture Collection (ATCC), and cultured as previously described [4]. CaSKi cells were grown in complete DMEM (Gibco-Invitrogen, Waltham, MA) supplemented with 10% heat inactivated foetal bovine serum (FBS) (Gibco- Invitrogen, Waltham, MA) and 1% of antibiotic/glutamine preparation (100 U/ml penicillin G, 100 U/ml streptomycin sulphate, and 2.9 mg/ ml of L-glutamine) (Gibco-Invitrogen, Waltham, MA). Immortalized normal human cervical keratinocytes, HCK1T, were kindly provided by Professor Tohru Kiyono (National Cancer Center Research Institute, Tokyo, Japan) and cultured as previously described [18]. HCK1T were grown in Keratinocyte serum-free media supplemented with 50μg/ml bovine pituitary extract and 5ng/ml human recombinant epidermal growth factor (Gibco-Invitrogen, Waltham, MA), 0.035mM CaCl2 and 1% of antibiotic preparation (100 U/ml penicillin G and 100 U/ml streptomycin sulphate) (Gibco-Invitrogen, Waltham, MA).

### Cell viability measurement

Cells were seeded overnight and treated the following day. At 72h, cell viability was determined by the 3-(4,5-dimethylthiazol-2-yl)-2,5-diphenyltetrazolium bromide (MTT) assay and half maximal inhibitory concentration (IC50) determined using GraphPad Prism v8.

### Simulated vaginal fluid (SVF) preparation and Alisertib exposure

SVF was prepared as previously described [19]. For experiments involving SVF exposure to Alisertib (Jomar Life Research, Caribbean park, VIC, Australia), Alisertib/DMSO was incubated with SVF before diluting in cell media.

### HPLC analysis of Alisertib

A stock solution of Alisertib (1 mg/mL) was prepared in 1% formic acid/acetonitrile for analysis via High Performance Liquid Chromatography (HPLC). The lambda max (λ max) of Alisertib was determined by scanning 200 μL of the stock solution from 200–650 nm on a MultiskanTM GO microplate spectrophotometer (Thermo Fisher Scientific, Waltham, MA) using 1% v/v formic acid in acetonitrile as a control. HPLC analysis was performed on a Shimadzu Prominence LC-20AT coupled to an SPD-M20A Diode Array Detector (DAD). Separation was achieved using a reverse phase C18 column (GraceSmart C18 150 × 4.6 mm, 3 μm, Grace Davidson, Australia; column oven set at 30°C) and a flow rate of 1.0 mL·min^-1^. The initial mobile phase consisted of 50% mobile phase B (0.1% v/v formic acid in HPLC grade acetonitrile (RCI Labscan, Thailand) and 50% mobile phase A (0.1% v/v formic acid in Milli-Q purified H_2_O). A linear gradient was applied: 0–0.25 minutes, 50% B; 0.25–4.75 minutes from 50% to 95% B; 4.75–6.75 minutes, 95% B. After 6.75 minutes, 50% B was run for 7 minutes to re-equilibrate the column in between analyses. A linear calibration curve was generated using 6 standard concentrations of Alisertib (3854.2 nM, 963.54 nM, 385.43 nM, 192.71 nM, 96.35 nM and 38.54 nM), and R^2^ was calculated. For experiments involving solubilizing Alisertib in isopropanol, 1 mg/ml (1% w/v) of Alisertib was prepared in 50% isopropanol for HPLC analysis.

### Liquid chromatography/mass spectrometry (LCMS) analysis of Alisertib

HPLC coupled to Electrospray Ionisation Mass Spectrometry (HPLC-ESI/MS) was conducted using a Shimadzu Nexera X2 UHPLC system with SPD-M30A DAD coupled to a Shimadzu LCMS-2020 single quadrupole mass spectrometer in order to confirm the m/z of Alisterib. Separation was achieved using a reverse phase C18 column (Shimadzu Shim-pack GISS C18, 150 mm x 2.1 mm, 3 μm; column oven set at 30°C) and a flow rate of 0.375 mL·min^-1^. A linear gradient was applied: 0.0–2.5 minutes, from 50% to 95% B; 2.5–3.5 minutes, 95% B. After 3.5 minutes, 50% B was run for 3 minutes to re-equilibrate the column in between analyses. Alisterib was constituted as above, added to a HPLC vial, placed in the autosampler where 10 μL was injected for analysis with data acquired in positive ion mode. Qualitative ESI-MS operating conditions were: nebulizer and drying gas (N_2_ via Peak Scientific NM32LA nitrogen generator), desolvation line temperature 250 °C, drying gas flow 15 L·min^−1^, nebulizer gas flow 1.5 L·min^−1^, heat block temperature 200 °C, interface voltage 4.5 kV with the MS scan range set from 200–650 m/z with an event time of 0.07 sec. Single Ion Monitoring (SIM) was also used with 519.25 m/z with an event time of 0.05 sec.

### Manufacturing intravaginal rings (IVRs) containing Alisertib

Mouse-sized molds were 3D printed on a Ultimaker 2+ printer (Cambridge, MA). The dimensions of the mold cavities were 4.2 mm diameter (including a 1.4 mm inner filled cylinder) x 1.4 mm depth. Biomedical grade SILASTIC MDX4-4210 silicone elastomer base (Dow Corning, Thailand) was used as a base material to manufacture the rings. Both the curing agent (1 part by weight) and the elastomer base (10 parts by weight) were heated to 80 °C to facilitate the curing process and to decrease silicone viscosity. Three sets of Alisertib rings were manufactured and tested; containing either 0.5%, 1% or 2% (w/w) Alisertib. The required amount of Alisertib powder was added to the silicone blend and mixed thoroughly using a stainless-steel spatula on a glass board for 10 minutes at room temperature. After mixing, the resulting blend was moved to a 1 ml syringe and injected through a 19-gauge medical needle with a removed bevel into the mold. The silicone was allowed to cure at room temperature overnight, then the mass of the rings recorded. A one-way ANOVA test was performed to identify any significant differences in weight between the three groups.

### Alisertib release profile assessment

IVRs were then suspended in scintillation vials containing release medium (50% isopropanol/water) to maintain sink conditions (Alisertib < 10% saturation) and hung using thin glass tubes to avoid floating. The vials were incubated in an orbital shaker at 60 round per minute (RPM), at 37 °C to simulate vaginal tract conditions. The medium was sampled every 24h for 5 days, and then every 48h for the remainder of the experiment for HPLC analysis. The cumulative release was calculated by integrating the area under the curve in the release graph (LabSolutions Analysis Data System, Shimadzu, Kyoto, Japan).

### Intravaginal ring implantation in mice

IVRs loaded with or without 1% ALISERTIB were pre-lubricated with K-Y Jelly (Johnson & Johnson, New Brunswick, NJ) before being implanted into the vaginal tract of 8 to 12-week- old FVB mice. On Day 7, mice were euthanized, and genital organs collected. Tissues were then embedded in paraffin and stained with haemotoxylin and eosin. Animal work was approved by the Griffith University Animal Ethics Committee (GU Ref No: MSC/06/16/AEC).

### Statistical analysis

Statistical analyses were done on the IBM SPSS Statistics 22 software. One-way ANOVA tests were performed where relevant and a 95% confidence interval (p-value < 0.05) was applied to compare controls and treated samples.

## Results

### Alisertib can be detected by HPLC and LC-MS analysis

**Fig 1:**
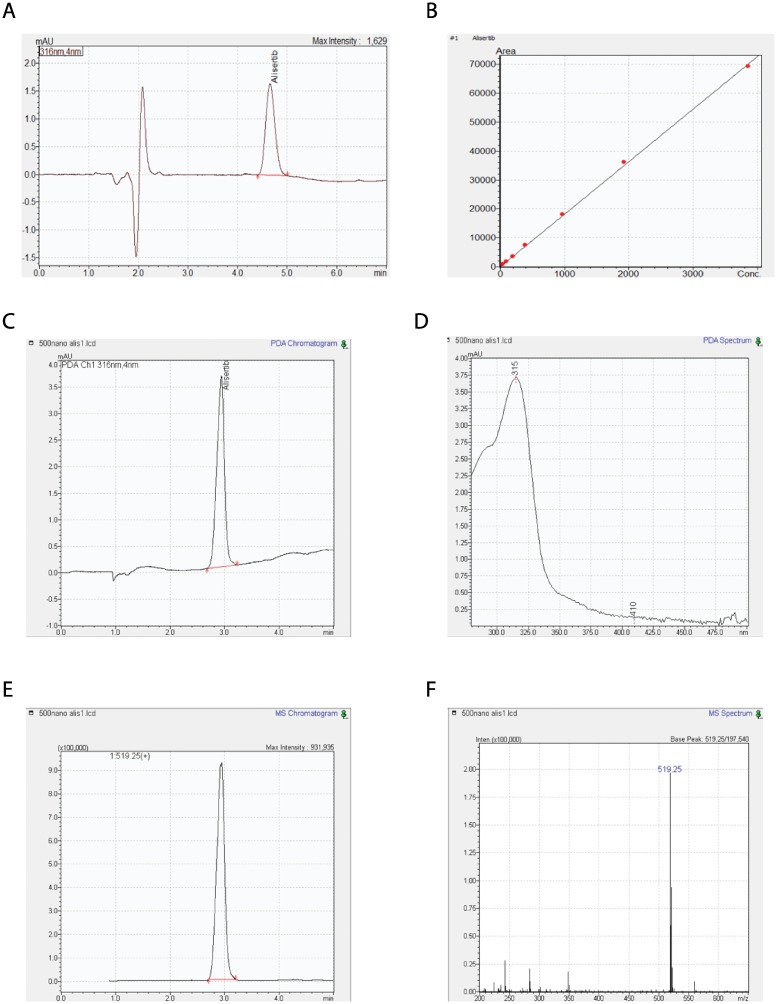
Alisertib can be detected by HPLC and LC-MS analysis. (a) DAD chromatogram of Alisertib standard injection for the developed HPLC method showing elution at 4.65 minutes (b) HPLC calibration curve for Alisertib from known concentrations (3854.2 nM, 963.54 nM, 385.43 nM, 192.71 nM, 96.35 nM and 38.54 nM) with an R^2^ value of 0.9999. Limit of Quantification (LOQ) = 21.37 nM, and Limit of Detection (LOD) = 7.05 nM. (c) DAD chromatogram of Alisertib standard injected for LCMS analysis at 316nm showing elution at 2.94 minutes (d) DAD spectrum of Alisertib standard injected for LCMS analysis at 2.94 minutes. (e) Electron ion chromatogram of the 519.25 m/z [M+H]^+^ for the Alisertib standard in acetonitrile showing elution at 2.94 minutes (f) Mass spectra of the 2.94 Alisertib minute peak showing the 519.25 m/z [M+H]^+^ ion in highest abundance. The chromatogram shows one large prominent peak eluting at 2.94 minutes.

Lambda max of the Alisertib standard was identified at 314–316 nm and the ion in greatest abundance under the peak of interest was 519.25 m/z [M+H]^+^. Thus, 316nm was used to quantify Alisertib via HPLC (Fig 1a). The mean AUC was calculated from triplicate injections and a calibration curve was generated (Fig 1b). Linear regression analysis was used to generate a line of best fit, which had an R^2^ value of 0.9999. This standard curve was used in the subsequent experiments to quantify Alisertib present in samples. LCMS analysis confirmed the m/z of the prominent Alisertib ion as 519.25 [M+H]^+^ which was consistent when Alisertib was constituted in both acetonitrile and isopropanol (Fig 1c). An electron ion chromatogram (EIC) for the 519.25+ m/z [M+H]^+^ ion after the Alisertib was injected is shown (Fig 1e and 1f). The LCMS mode confirmed the reliability of the developed method in detecting Alisertib in isopropanol.

### HPV-transformed cervical cancer cells were sensitive to Alisertib treatment and Alisertib bioactivity unchanged upon exposure to simulated vaginal fluid and high temperature conditions

**Fig 2:**
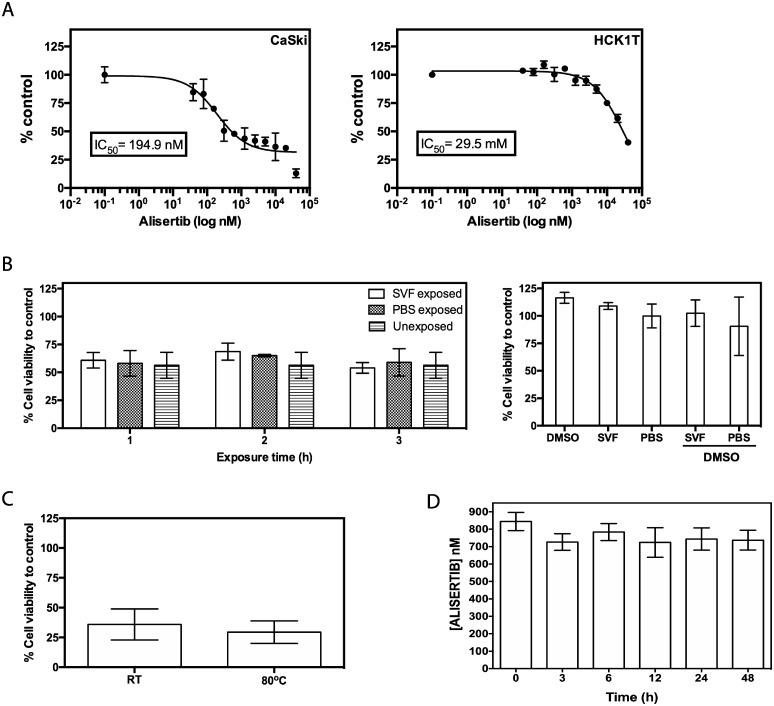
HPV-transformed cervical cancer cells were sensitive to ALISERTIB treatment and bioactivity unchanged upon exposure to simulated vaginal fluid and high temperature conditions. (a) CaSki and HCK1T cells were treated with ALISERTIB at doses ranging from 40mM to 40nM in a 96 well plate. Cell viability was measured by the MTT assay at 3- and 6-days’ post-treatment for CaSki and HCK1T, respectively. A dose dependent curve was generated using Graphpad Prism v8 to determine IC50 values for each drug-treated cell line. Data points are representative of the mean ± standard deviation (SD) (n = 3). CaSki cells were treated with 200nM of ALISERTIB (b) either unexposed or exposed to simulated vaginal fluid (SVG) or PBS for the indicated length of time or (c) subjected to heating at 80°C or left at room temperature (RT) for 30min. After 72h, cell viability was measured by the MTT assay. Data is representative the mean ± SD percentage cell viability of treated to untreated (control) cells. (d) 1 mg/ml ALISERTIB (1% w/v) was dissolved in 50% isopropanol/H2O over time at 37 °C before measuring the concentration of ALISERTIB by HPLC. Data is representative of mean ± SD.

We previously showed that the presence of HPV major oncogene, E7, sensitized cervical cancer cells to killing by Alisertib (4). To assess the optimal dose for Alisertib to be therapeutically effective, the IC50 of Alisertib of CaSKi cells (194.9 nM), an HPV+ cervical line, was determined. Compared to the IC50 of HCK1T cells (IC50 = 29.5 mM), HPV-negative (-) immortalized primary cervical keratinocytes, CaSki cells were roughly 100-fold more sensitive to Alisertib treatment (Fig 2a). Next, we assessed whether Alisertib would retain its biological activity in the often hydrophilic and low pH vaginal microenvironment. To simulate these conditions, we incubated Alisertib in SVF before treating CaSki cells. SVF is commonly used to test vaginal rings [20]. Cytotoxicity of SVF-exposed Alisertib was comparable to that of Alisertib itself or exposed to PBS, irrespective of incubation time, suggesting that Alisertib is able to maintain its biological activity in SVF (Fig 2b). As IVR manufacturing and Alisertib embedding process involved high temperature conditions, we confirmed that heating Alisertib to 80 °C for 30 min did not affect its biological activity (Fig 2c). Another important facet to consider is the stability of Alisertib during the downstream extraction process for quantifying the Alisertib in isopropanol. HPLC analysis revealed minimal degradation of Alisertib in these conditions, even after 48h (Fig 2d). Overall, we show that Alisertib bioactivity remains unchanged upon its exposure to SVF and high temperatures and its stability is maintained in isopropanol over time.

### Alisertib is released from intravaginal rings in a controlled and sustained kinetic over 3 weeks *in vitro*

**Fig 3:**
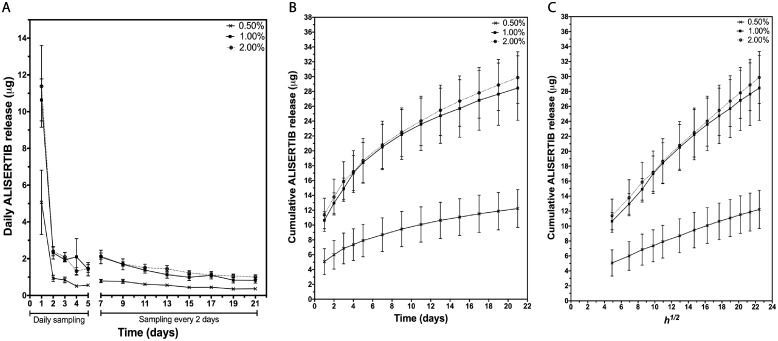
Alisertib is released from intravaginal rings in a controlled and sustained kinetic over 3 weeks *in vitro*. Mouse-sized rings loaded with various concentrations of ALISERTIB (0.5%, 1%, 1.5% and 2%) w/w were suspended in scintillation vials containing the release medium before incubating the rings individually in 50% isopropanol/water. Rings were then left in an orbital shaker at 37 °C and medium sampled every 24h for the first 5 days, and then every 48h for the remaining weeks. Mass of ALISERTIB release was measured by HPLC. (a) Daily ALISERTIB release versus time (days) (b) cumulative ALISERTIB release versus time (days) and (c) cumulative ALISERTIB release versus square root time (h^1/2^) are plotted. Data is representative of the mean ± standard error of mean (SEM) of triplicate measurements.

To investigate Alisertib release kinetics, intravaginal rings were suspended in a scintillation vial in an appropriate volume of release medium and in vitro release profile from the rings into 50% isopropanol/water was measured over 21 days. Alisertib-embedded rings (0.5%, 1% or 2% (w/w) Alisertib) were manufactured with no significant weight differences (ANOVA test, F = 1.03, p > 0.05). Release media was replaced, and Alisertib concertation measured every 24h with fresh media for the initial 5 days and then every 48h after 5 days. As expected, a high amount of Alisertib was released on Day 1 and that the cumulative Alisertib release profile was gradual over 21 days (Fig 3). Importantly, Alisertib release kinetics obeyed root time (t^1/2^) kinetics when plotted on a linear cumulative release versus square-root time profile (R^2^: 0.5% = 0.9963, 1% = 0.9883, 2% = 0.9972). Notably, Alisertib release profiles for both the 1% and 2% Alisertib rings were identical most likely due to over-saturation of Alisertib for concentrations above 1% (w/w).

### Alisertib-loaded intravaginal rings did not cause any local inflammation in the vaginal tract

**Fig 4:**
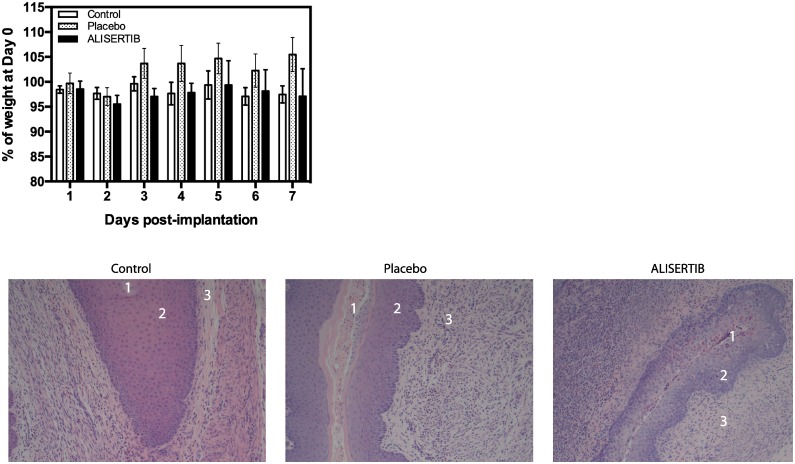
Alisertib-loaded intravaginal rings did not induce overt inflammation in the mouse vaginal tract. FVB mice were either not implanted (control) or implanted with rings containing 1% (w/w) Alisertib in the vaginal tract. Intravaginal rings without Alisertib were used as the placebo. Mice were weighed daily over a week. Data is representative of the percentage of the mean weight at Day 0 ± standard error of mean (SEM) from five mice. Tissue sections of the mice genital tract were examined to any noticeable signs of abscess or inflammation in the (1) vaginal lumen, (2) epithelium and (3) lamina propria regions of the tissue. Tissues were collected at Day 7 and stained with H&E.

Finally, we wanted to address the general safety of administering this delivery device in the mouse vaginal tract. Previous studies that implanted vaginal rings in mice used sutures to secure the rings in place [21, 22]. No suturing was done in our experiments to minimize procedure-related disturbance to the vaginal tract. All rings were confirmed to be in the vaginal tracts on Day 3 or 4, but not after Day 7, most likely due to displacement of the rings out of the vaginal tract over time. Given that the highest amount of ALISERTIB is released within the first few days (Fig 3), we decided that it was important to assess the effect of this short-term high loading dose on the implanted genital region. We confirmed that there is no notable local inflammation associated with our implants, nor any effect on the weight of the mice (Fig 4).

## Discussion

In this study, we developed a potential topical vaginal delivery platform of Alisertib for the potential treatment of cervical cancer. Although, topical delivery to the cervix is feasible, it was necessary to explore and optimize biological, chemical and technical aspects of the chosen delivery platform. Oral administration of Alisertib can have severe adverse clinical effects following phase I/II trials (See review by Tayyar et al., [23]). In this study, we have chosen silicone intra-vaginal rings for their ability to deliver hydrophobic drugs over an extended period of time based on the reliability achieved by those currently commercially available [8, 20]. As Alisertib is a highly hydrophobic compound (logP = 5.74, predicted using MarvinSketch Software, ChemAxon), it was logical to first begin with testing the ability to incorporate it in silicone, the historical polymer of choice.

We first developed a sensitive HPLC method for the detection and quantifying of Alisertib in its solutions and performed LCMS analysis to ensure the identity of the standard. We then ensured that Alisertib was able to retain activity in the vaginal tract by simulating the same conditions. Interestingly, the biological efficacy of Alisertib showed high tolerance to all conditions related to exposure to SVF (i.e. acidic conditions) and the process of embedding the drug into silicone rings (i.e. high temperature conditions) (Fig 2b and 2c). An important consideration when studying intra-vaginal rings is measuring and optimizing their drug release profiles. The medical grade silicone elastomer used in fabrication is limited to external use or short-term implant applications over 29 days, hence why Alisertib release profile was initially tested over 21 days (Fig 3). We were also interested in testing the safety of a high loading dose followed by a continuous release over 3 weeks as we believe this will lead to a more rapid initiation of the biological effects of Alisertib. Here we demonstrate the “burst effect” phenomenon with Alisertib (Fig 3), typically seen in matrix type vaginal rings [20]. This was expected and observed in all Alisertib ring concentrations, where a relatively high amount of drug was released over the first 2 days. The burst effect could easily be overcome by employing reservoir-type or shell-type rings. However, we anticipate beneficial anti-cancer effect due to the initial high loading dose. We also showed that Alisertib-loaded rings did not result in any overt inflammation or anatomical disturbances over the first week of application *in vivo* (Fig 4). These preliminary results encourage us to take this platform forward for future efficacy testing and longer treatment cycles in orthotopic patient-derived xenograft cervical cancer *in vivo* models.

In conclusion, we show that Alisertib is a suitable candidate for vaginal delivery using intra-vaginal rings, and the use of this molecule for topical treatment is justified. We developed a sensitive HPLC method for the quantification of Alisertib in its solutions then tested the release of the manufactured rings over 21 days. *In vitro*, the manufactured rings achieved relatively reasonable release kinetics over 21 days and exhibited the “burst effect” with root time kinetics over an extended period of time. Importantly, Alisertib-loaded rings did not cause any short-term local inflammation within the mouse vaginal tract.
